# Decrementing Evoked-potential Propagation Map Defines the Ventricular Tachycardia Isthmus

**DOI:** 10.19102/icrm.2021.120119S

**Published:** 2021-01-15

**Authors:** Lim Ven Gee, Faizel Osman, Sandeep Panikker, Shamil Yusuf, Tarvinder Dhanjal

**Affiliations:** ^1^Department of Cardiology, University Hospital Coventry & Warwickshire NHS Trust, Coventry, England; ^2^University of Warwick Medical School, Gibbet Hill, Coventry, England

**Keywords:** Catheter ablation, decrementing evoked potentials, high-density mapping, propagation map, ventricular tachycardia

We present a 65-year-old man with ischemic cardiomyopathy, prior myocardial infarction with mid-left anterior descending coronary artery occlusion, and an implantable cardioverter-defibrillator (ICD) who presented in December 2019 with ventricular tachycardia (VT) storm, receiving several ICD shocks. He was commenced on oral amiodarone; however, in February 2020, he presented with further VT and appropriate shocks. He underwent VT ablation under conscious sedation with antegrade mapping of the left ventricle (LV) using the Advisor™ HD Grid Mapping Catheter, Sensor Enabled™ combined with the steerable Agilis™ sheath. The sinus rhythm LV substrate map confirmed extensive septal and apical scar as shown in **Figure 1A**. Within the dense scar region (< 0.5 mV), a low-voltage conduction channel (CC) at the highlighted high-density grid position was identified (white arrow in **Figure 1A**). Sinus rhythm low-amplitude ventricular activities were identified at the anterior border zone (BZ) of the CC; however, as shown in **Figure 1C**, they were buried within the QRS complex.

We routinely performed sensed extras (coupling interval: 400 ms) from the right ventricular apex to identify decrementing evoked potentials (DEEP), as shown in **Figure 1D**. The propagation map of the DEEP potentials is shown in the series of images in **Figure 1E**, highlighting a figure-of-eight activation wavefront starting at the inferior BZ that extended around the scar and entered the CC at the anterior BZ. Ablation at 50 W using the TactiCath™ ablation catheter (lesions shown in **Figure 1B)** targeting the CC entrance site successfully eliminated the CC and homogenized the scar. The postablation propagation map of the DEEP potentials confirmed no further late activation within the scar, with the activation wavefront traversing around the scar from the inferior LV to the anterior LV **(Figure 1F and Video 1)**. Postprocedure, no clinical or nonclinical VT was inducible and the patient has remained free of ICD therapy.

## Figures and Tables

**Figure 1:**
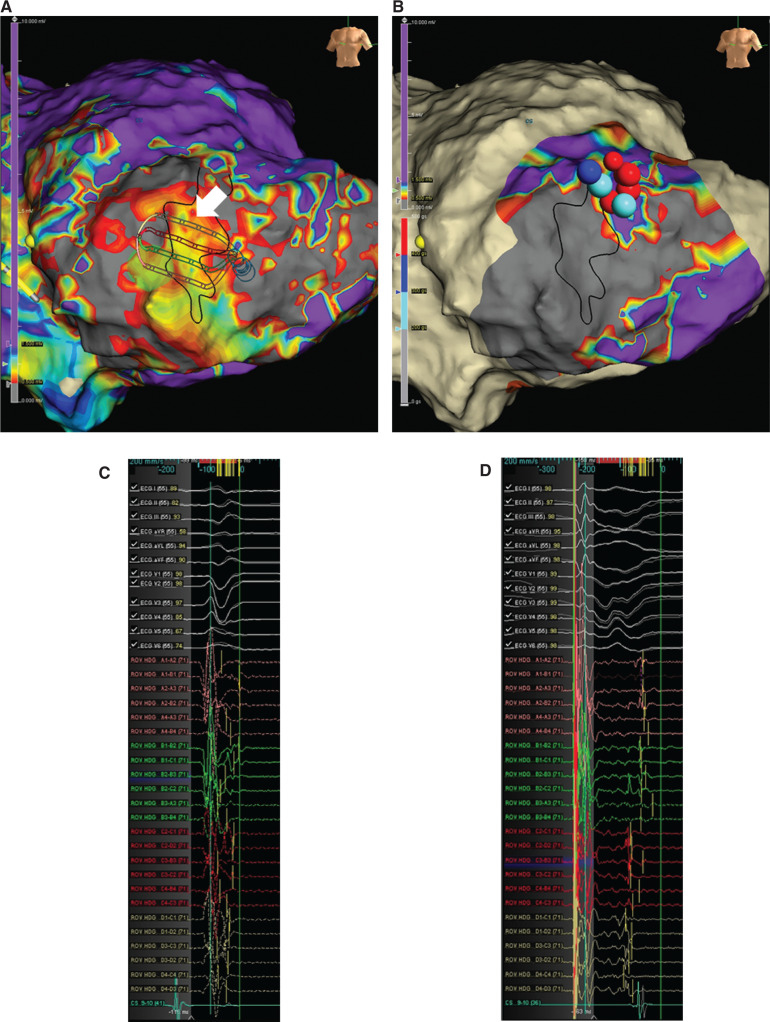
A DEEP propagation map was used to define the VT isthmus. **A:** Substrate map with high-density grid overlaying the VT isthmus. **B:** Substrate map postablation of entrance into the VT isthmus. **C:** Late potentials in sinus rhythm identified within the VT isthmus. **D:** DEEP signals with sensed extras from right ventricular apical pacing.

**Figure 1:**
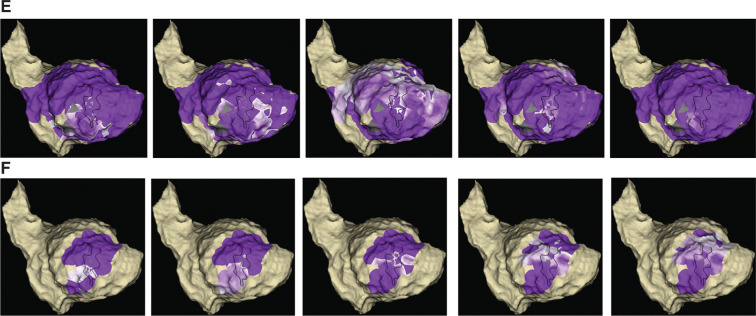
**E:** Propagation map of DEEP signals preablation. **F:** Propagation map of DEEP signals postablation.

**Video 1.** First two wavefronts show propagation map of the DEEP potentials preablation and the second two wavefronts showing propagation map of the DEEP potentials postablation.

